# Male great tits assort by personality during the breeding season

**DOI:** 10.1016/j.anbehav.2017.04.001

**Published:** 2017-06

**Authors:** Katerina V.-A. Johnson, Lucy M. Aplin, Ella F. Cole, Damien R. Farine, Josh A. Firth, Samantha C. Patrick, Ben C. Sheldon

**Affiliations:** a University of Oxford, Department of Zoology, Edward Grey Institute, Oxford, U.K.; b University of Oxford, Department of Experimental Psychology, Oxford, U.K.; c University of Konstanz, Department of Collective Behaviour, Max Planck Institute for Ornithology, Konstanz, Germany; d University of Liverpool, Department of Earth, Ocean & Ecological Sciences, Liverpool, U.K.

**Keywords:** exploration behaviour, great tit, *Parus major*, personality, social networks

## Abstract

Animal personalities can influence social interactions among individuals, and thus have major implications for population processes and structure. Few studies have investigated the significance of the social context of animal personalities, and such research has largely focused on the social organization of nonterritorial populations. Here we address the question of whether exploratory behaviour, a well-studied personality trait, is related to the social structure of a wild great tit, *Parus major*, population during the breeding season. We assayed the exploration behaviour of wild-caught great tits and then established the phenotypic spatial structure of the population over six consecutive breeding seasons. Network analyses of breeding proximity revealed that males, but not females, show positive assortment by behavioural phenotype, with males breeding closer to those of similar personalities. This assortment was detected when we used networks based on nearest neighbours, but not when we used the Thiessen polygon method where neighbours were defined from inferred territory boundaries. Further analysis found no relationship between personality assortment and local environmental conditions, suggesting that social processes may be more important than environmental variation in influencing male territory choice. This social organization during the breeding season has implications for the strength and direction of both natural and sexual selection on personality in wild animal populations.

## Highlights

• We assess whether a great tit breeding population is structured by personality.
• Network analyses were conducted on a 6-year data set from this wild bird population.
• Males show positive assortment, nesting nearer to similar personalities (bold/shy).
• This assortment was not found to be related to local environmental variation.
• We discuss implications for natural and sexual selection on personality in the wild.

Recent years have seen a growing interest in understanding the causes and consequences of animal ‘personality’ (Dall et al., 2004, Sih et al., 2004). Although complete behavioural plasticity might be expected to be the optimum strategy, individuals are typically limited in their range of behavioural expression, with variation observed in the population (Sih et al., 2004). These interindividual behavioural differences, which are consistent over time and correlated across different contexts, are referred to as behavioural syndromes or personality traits (Wolf & Weissing, 2012). They often have a genetic basis (Drent et al., 2003, van Oers et al., 2005), are linked to a range of life history traits (Groothuis & Carere, 2005) and can have important fitness consequences (Dingemanse et al., 2004, Smith and Blumstein, 2008). Behavioural phenotypes may therefore be subject to natural and sexual selection (Dingemanse, Kazem, Réale, & Wright, 2009).

An understanding of the social context of personality is imperative since an organism's social environment represents a key component of selection (Bergmüller and Taborsky, 2010, Krause et al., 2009, Oh and Badyaev, 2010, Réale et al., 2010). Indeed, an individual's fitness is influenced not only by its own phenotype but also by the phenotype of the individuals with which it interacts (Farine and Sheldon, 2015, Formica et al., 2011, West-Eberhard, 1979, Wolf et al., 1999). The importance of social structure has often been overlooked as much work assumes a randomly mixed population (Farine et al., 2015). However, social interactions typically occur nonrandomly within populations, with individuals varying in their number and strength of connections (Croft, James, & Krause, 2008). Several studies across vertebrate taxa (including fish, birds and mammals) have found that individuals are nonrandomly distributed within social networks with respect to their personality (Aplin et al., 2013, Best et al., 2015, Carter et al., 2015, Croft et al., 2009, Snijders et al., 2014). This assortment may have consequences for social functioning (Wolf & Krause, 2014) and for the strength and direction of selection on behavioural phenotypes (Croft et al., 2009, Krause et al., 2010, Wilson et al., 2013).

While empirical studies exploring the relationship between social structure and personality in wild populations have demonstrated that personality can be an important phenotypic trait influencing social organization, such studies have largely been restricted to foraging groups (Aplin et al., 2013, Best et al., 2015, Carter et al., 2015, Croft et al., 2009). By contrast, little is known about social network structure with respect to personality in territorial populations, such as during the breeding season of many songbirds. Social interactions at this time are likely to be fundamentally different, with less influence of interactions related to foraging (e.g. social information and predator avoidance) and more influence of interactions related to reproduction (e.g. mating opportunities and male–male competition). The only previous investigation of the relationship between personality and social structure in a territorial breeding population showed, via automated tracking, that slow-exploring male great tits, *Parus major*, tended to occupy less central network positions (Snijders et al., 2014).

In this study, we used data from a wild great tit population spanning 6 years to examine whether individuals show spatial assortment by personality type during the breeding season. In particular, we assessed exploration behaviour (the degree to which individuals explore a novel environment) since this is commonly used as a proxy for the reactive–proactive axis, including in our study species (Quinn, Patrick, Bouwhuis, Wilkin, & Sheldon, 2009). This behavioural axis contrasts shy, slow-exploring individuals with bolder, fast-exploring individuals (Quinn, Cole, Bates, Payne, & Cresswell, 2012). The continuous variation along this axis is hypothesized to result from the inherent trade-off between predator-averse behaviour favouring survival and risk-prone behaviour prioritizing productivity (Biro and Stamps, 2008, Wolf et al., 2007). Previous work on our study population has demonstrated positive assortment among males by this measure of personality type in winter foraging flocks (Aplin et al., 2013). This suggests that shy birds may actively modify their social environment by avoiding bolder, more aggressive individuals (Aplin et al., 2013). Indeed, aggressive interactions between males are more intense during the territorial period and aggression is known to be positively correlated with exploratory behaviour in this species (Carere, Drent, Privitera, Koolhaas, & Groothuis, 2005). We therefore hypothesized that birds may also demonstrate positive assortment by personality with respect to their choice of breeding location.

## Methods

### Study System

Data were collected from a wild great tit population at Wytham Woods, Oxfordshire, U.K. (51°46′N, 01°20′W), which is a mixed deciduous woodland of 385 ha, bordered by farmland. This population is part of a continued long-term breeding survey, which began in 1947 and monitors the location and identity of nesting pairs (Savill, Perrins, Kirby, & Fisher, 2010). The majority of great tits at this study site have a unique metal leg ring and they generally nest in one of the 1018 nestboxes in the woodland (Aplin, Farine, Morand-Ferron, Cockburn, Thornton, & Sheldon, 2014). The population exhibits fission–fusion dynamics over autumn and winter, whereas the social structure changes in the breeding season (Psorakis, Roberts, Rezek, & Sheldon, 2012). During this period, typically from March to June, socially monogamous pairs hold and defend territories around the nestboxes (Hinde, 1952).

### Personality Assays

An individual's position along the shy–bold personality axis was estimated using an assay of exploration behaviour in a novel environment. These assays were first conducted in 2005 and have been carried out in subsequent years according to the same methodology (Quinn et al., 2009). Great tits were captured via mist netting during winter and kept in individual indoor aviaries overnight. Birds were assayed individually the following morning for 8 min in a novel environment room with five perches. The frequency and location of their movements were recorded with a handheld computer. These observations were incorporated into a principal component analysis to generate an exploration score for each bird on a continuous scale, such that individuals visiting each of the five perches and each of the five areas, and with a greater frequency of hops and duration of flights, were assigned a higher exploration score (Quinn et al., 2009). Exploration behaviour was moderately repeatable within and between our assaying seasons (Quinn et al., 2009) and has been shown to correlate with a wide range of functional behaviours in our wild population (Aplin et al., 2014a, Aplin et al., 2013, Cole and Quinn, 2012, Cole and Quinn, 2014, Patrick et al., 2012, Quinn et al., 2011, Quinn et al., 2012).

### Social Networks During Breeding Season

Associations between individuals were inferred based on the spatial proximity of occupied nestboxes. By connecting individuals (nodes) via associations (edges), social network analysis provides a means to assess fine-scale population structure (Farine and Whitehead, 2015, Krause et al., 2010). Network analyses and all associated statistics were performed in R 3.2.3 (R Development Core Team, 2015). Networks were constructed for six consecutive breeding seasons from 2005 to 2010. To account for local breeding densities, we classified individuals as associated with their *k* nearest neighbours for a range of values of *k* (where *k* = 3, 5 or 7). As weighted networks provide more robust estimates of assortment (Farine, 2014), we also assigned weights to each edge $e_{ij}$, where $e_{ij}=\frac{1}{\text{ln}\left(d\right)}$ and d is the Euclidean distance between the nestboxes in which individuals i and j are breeding (but where $e_{ij}=0$ if j is not a *k*th nearest neighbour of i). This measure of proximity is particularly relevant, as the slope of decline in extrapair paternity with distance follows a log-linear relationship (Hadfield, 2012) and so it is reasonable to assume that territorial interactions may occur on a comparable spatial scale. Since nearest-neighbour networks are not necessarily symmetrical (as one individual may be another's nearest neighbour but not vice versa), this method generates directed networks. For comparison, networks were also constructed using the Thiessen polygon method to approximate neighbours. Associations were assigned based on which individuals shared a boundary when Thiessen polygons were created around each occupied nestbox to predict territories (Schlicht, Valcu, & Kempenaers, 2014). The polygons were generated using the packages spatstat (Baddeley & Turner, 2005), spdep (Bivand, 2016), maptools (Bivand & Lewin-Koh, 2012) and rgdal (Bivand, Keitt, Rowlingson, & Pebesma, 2012). These Thiessen polygon networks are undirected, but individuals can vary in their number of neighbours based on the geometry of their territory. We use the term neighbour throughout to describe any two individuals that are connected in a network, although with the nearest-neighbour method, this may not necessarily equate to sharing a territory boundary.

Separate networks for males and females were generated and analysed independently. Additionally, social networks were constructed for one section of the woods, incorporating Marley Wood and Marley Plantation (subsequently referred to as Marley). This easterly area of Wytham Woods is 51 ha in size and contains 317 nestboxes. This region was selected as it has been the focus of previous research effort and so has a particularly high proportion of breeding individuals with an assigned personality score (Patrick et al., 2012, Szulkin et al., 2012), which is important for maximizing the resolution of the networks (Farine and Strandburg-Peshkin, 2015, Silk et al., 2015).

### Statistical Analyses

Data from 2005 to 2010 were incorporated in this study since the most extensive personality data were available for this period. Subsequent years were omitted given their low proportion of breeding individuals with a known personality. For each year's network, assortment with respect to personality was calculated using Newman's assortment score (Newman, 2003). This score measures the correlation between an individual's phenotype and that of its associates and is a commonly used statistic to detect phenotypic structure in social networks (Farine & Whitehead, 2015). The assortnet package was used which allows assortativity to be measured for continuous traits with both binary and weighted networks (Farine, 2014). Assortativity was also calculated separately for the networks restricted to Marley. Since only personality-typed individuals were relevant to this analysis (Table 1), the power of the data set was limited by the proportion of breeding individuals whose personality had been assayed (approximately 0.3 across all of Wytham Woods for both males and females and in Marley 0.4 for males and 0.5 for females).

**Table 1:** Summary data for the study population

Location	Year	Breeding pairs	Personality-typed males		Personality-typed females	
			Number	Proportion	Number	Proportion
Marley	2005	120	46	0.383	53	0.442
	2006	93	47	0.505	51	0.548
	2007	107	51	0.477	69	0.645
	2008	108	45	0.417	48	0.444
	2009	79	38	0.481	45	0.570
	2010	93	32	0.344	47	0.505
Wytham	2005	493	99	0.201	107	0.217
	2006	432	142	0.329	144	0.333
	2007	481	159	0.331	203	0.422
	2008	470	140	0.298	168	0.357
	2009	324	106	0.327	122	0.377
	2010	381	94	0.247	116	0.304

The table shows the total number of breeding pairs each year within Wytham Woods and the Marley region of the woods, and the number and proportion of breeding males and females with known personality.

Assortativity analyses were conducted both for individual years and across all years. For each year, a network was constructed (for males and females separately) using either the *k*-nearest-neighbour algorithm or the Thiessen polygon method to assign associations. The networks were restricted to only include nodes representing individuals with a personality score. The observed assortment was calculated, and the number of links in the network was recorded. To assess assortativity relative to null expectation, while also accounting for the nonindependence of social network data, 1000 node-based randomizations were performed on each year's observed network. Under this null model, an individual's network position is assumed to be independent of personality type, while preserving network structure and personality scores. A node permutation approach is relevant in this case because we have high certainty of the network structure (Croft et al., 2009). The *P* value was then calculated by comparing the observed assortment score to the distribution generated from the 1000 randomizations to determine whether the pattern of assortment was significantly different from that predicted by the null model. To assess the average assortment across years, the means of the observed and null assortment scores were calculated, weighted by the number of links in each year's network. Some individuals bred in more than 1 year and so occasionally there were duplicated links between the same two individuals over multiple years, resulting in pseudoreplication in the combined data set (2005–2010). Although any pseudoreplication was held consistent in the null model (and therefore should not impact the estimated significance value), the analysis was also rerun by conducting 1000 randomizations on the combined data set such that repeated associations between years were randomly deleted prior to network construction.

In addition to the influence of interindividual behavioural differences, ecological factors can also contribute to a nonrandom spatial distribution of behavioural phenotypes. For example, shy personality types might breed in lower quality habitats, thus generating phenotypic assortment via a habitat–behaviour covariance. To determine the extent to which local environmental conditions influence social structure during the breeding season, repeatability was calculated with respect to the personalities of individuals occupying the nestboxes. A generalized linear mixed-effects model (GLMM) was used to estimate repeatability (Nakagawa & Schielzeth, 2010). This tested whether certain personality types were more likely to reoccur in the same nestbox, which would suggest an environmental effect. This model was implemented using the MCMCglmm package (Hadfield, 2010), with personality score as the dependent variable, sex as a fixed effect and year and nestbox as random effects. Some individuals bred repeatedly in the same nestbox across multiple years (representing approximately 14% of nestbox occupancies). To account for this statistically, the data set was subsampled using 1000 randomizations so that only one breeding attempt per individual in a given nestbox was included in the model each time.

A further important consideration was whether different personalities nest in areas of differing population densities. For instance, if bold (or shy) individuals choose to nest in regions with a higher density of occupied nestboxes, this could lead to a nonrandom distribution of behavioural phenotypes within the social network. To investigate the relationship between an individual's personality and the density of neighbouring territory holders, a GLMM was constructed with node strength (sum of edge weights as a measure of proximity to neighbours) as the dependent variable. For each node, the strength was calculated with the igraph package (Csardi & Nepusz, 2006) and used as a proxy for the local population density that an individual experiences. Personality and sex were fitted as fixed effects while year, nestbox and individual were included as random effects in the model. If there were repeated associations between years, only one was randomly selected in each of the 1000 randomizations before calculating node strength.

### Ethical Note

All work was subject to review by the ethical committee at the Department of Zoology (University of Oxford) and adhered to U.K. standard requirements. Birds were caught, tagged and ringed by British Trust for Ornithology licence holders, and held under Natural England licences.

## Results

**Figure 1:**
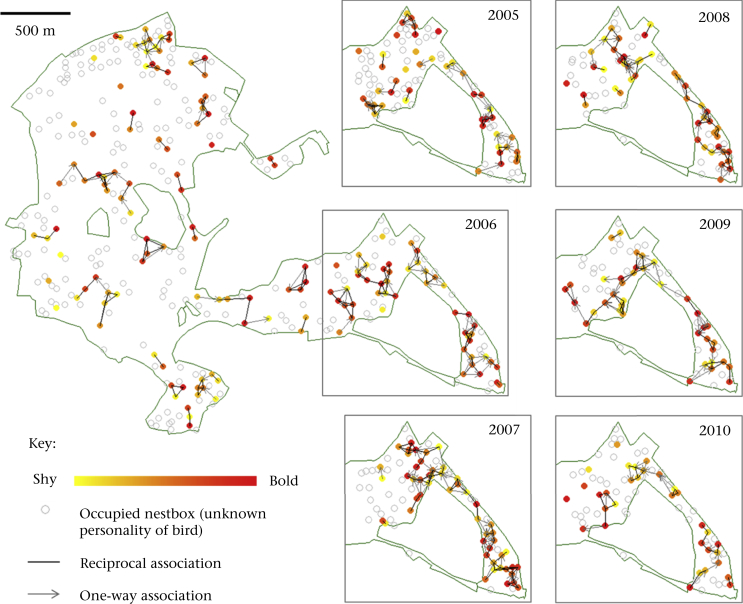
Male social networks (five nearest neighbours) in Wytham's great tit population during the breeding season. A representative network for Wytham is depicted (2006), along with the Marley networks for each year (2005–2010). Coloured nodes denote individuals with a personality score and open circles mark nestboxes occupied in that year but where the male's personality is unknown. Black lines denote a two-way association (both each other's nearest neighbours), whereas grey arrows depict the direction of one-way associations. For these directed networks, 70–80% of all links are reciprocal. The equivalent networks for females are shown in Fig. A1 in Appendix 1.

**Figure 2:**
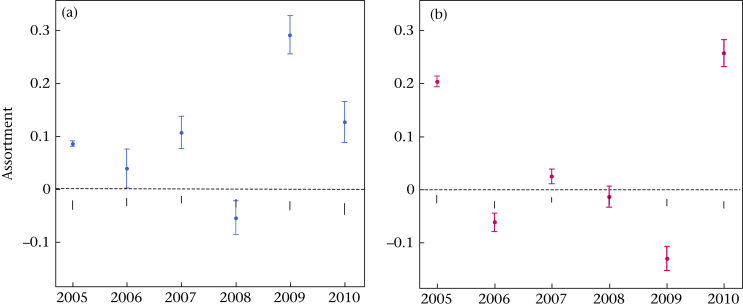
Assortment with respect to personality among (a) male and (b) female great tits in Marley during the breeding season. Circles mark the observed assortment score for each year (based on 5-nearest-neighbour networks), plotted with the standard error calculated from jackknife simulations. Vertical black lines denote the 95% confidence intervals generated under the null model (i.e. the values of assortment that are expected by chance given our data).

Within Marley, the region of the woods with the highest proportion of individuals with a known behavioural phenotype, there was significant positive assortment by personality among males (Fig. 1), as revealed by the *k*-nearest-neighbour method. This was true for all values of *k* (Table 2). Male assortment was also evident when conducting the analyses on binary rather than weighted networks (Table A1 in Appendix 2) and when there were no duplicated associations present in the data set (Table A2 in Appendix 2). Similar patterns of assortment were observed when considering each year individually (Table A3 in Appendix 2). In fact, males in all years except 2008 were positively assorted by personality type, with the observed assortment (±SE) lying outside the 95% confidence intervals generated from the node-based randomizations (Fig. 2a). In contrast, there was no evidence of consistent assortment across years among females within the nearest-neighbour networks (Table 2), although in 2005, 2007 and 2010, females also displayed a tendency towards positive assortment (Fig. 2b).

**Table 2:** Results of assortativity analyses on weighted social networks within Marley using the nearest-neighbour (NN) method

Network type	Male					Female				
	No. of links	Assortment		Effect size	*P*	No. of links	Assortment		Effect size	*P*
		Observed	Permuted				Observed	Permuted		
3-NN	60.5	0.140	−0.034	0.174	**0.020**	93.2	0.004	−0.024	0.028	0.606
5-NN	102.7	0.092	−0.027	0.120	**0.030**	153.2	0.041	−0.024	0.065	0.130
7-NN	141.8	0.108	−0.027	0.135	**0.004**	212.0	0.046	−0.024	0.070	0.066

The observed and null assortment scores (averaged across all 6 years) are based on weighted networks, with values in bold indicating significance at *α* = 0.05. The permuted assortment represents the mean of 1000 randomizations according to the null model. Average distances between nearest neighbours are shown in Table A4 in Appendix 2.

When nesting locations for the entire woodland were included in the nearest-neighbour analysis, males showed significant positive assortment by personality type when seven neighbours were considered (but not five or three neighbours), while there was no significant assortment among females (Table A5 in Appendix 2). In comparison to the nearest-neighbour method, there was no evidence of male or female assortment by personality when network associations were predicted using Thiessen polygons either in Marley or throughout the woods (Table 3).

**Table 3:** Results of assortativity analyses on social networks within Marley and throughout Wytham Woods using the Thiessen polygon method

Network type	Male					Female				
	No. of links	Assortment		Effect size	*P*	No. of links	Assortment		Effect size	*P*
		Observed	Permuted				Observed	Permuted		
Marley (weighted)	99.7	−0.017	−0.031	0.014	0.796	149.3	0.042	−0.028	0.070	0.122
Marley (binary)	99.7	−0.001	−0.029	0.029	0.568	149.3	0.039	−0.027	0.066	0.142
Marley (no duplicates)	96.0	−0.015	−0.032	0.017	0.755	143.2	0.031	−0.024	0.055	0.238
Wytham (weighted)	235.0	0.000	−0.013	0.013	0.694	320.0	0.025	−0.010	0.035	0.258

The observed and null assortment scores (averaged across all 6 years) are shown for both weighted (including duplicated associations removed) and binary networks. The permuted assortment represents the mean of 1000 randomizations according to the null model.

There was no evidence that certain nestboxes were consistently occupied by individuals of certain personality types since the average repeatability was negligible (MCMCglmm: repeatability coefficient = 0.004, 95% confidence interval = 0.000–0.018, *N* = 640 nestboxes). Additionally, local population density was not related to personality (MCMCglmm: coefficient = 0.000, *P* = 0.921).

## Discussion

By combining personality assays with social network structure, we demonstrate that male, but not female, great tits are positively assorted during the breeding season with respect to their personality. Thus, males are more likely to nest near other males of similar personality than expected by chance, while dissimilar personality types are more spatially segregated. Calculating the mean assortment over years, in addition to analysing individual years, increased the power of the data set and thus the ability to detect assortment. This pattern of assortment in the nearest-neighbour networks suggests that individual decisions about where to breed may involve factors relating to their social environment. Our findings therefore highlight the potential role of behavioural phenotypes in shaping social structure, which may in turn influence selection experienced by individuals of territorial species.

Significant positive assortment with respect to personality was also observed among males, but not females, in the winter social networks of this great tit population, perhaps because shy males seek to avoid interactions with bolder, more aggressive individuals (Aplin et al., 2013). Interestingly, positive assortment is a widespread characteristic of human social networks (often referred to as homophily) with respect to many different attributes, including personality (McPherson, Smith-Lovin, & Cook, 2001). The effect of behavioural traits on population structuring may be prevalent in animal societies (Aplin et al., 2013, Best et al., 2015, Carter et al., 2015, Croft et al., 2009, Snijders et al., 2014), particularly among group-living species, and future research should explore this further.

Our finding that breeding males show significant spatial assortment by personality type in Marley is unlikely to be a property of this region of the study site. Rather, it probably reflects the fact that the ability to detect assortment within a network is dependent on the number of individuals with a known phenotype in the population since this dictates the density of links in the network. Indeed, a recent study found that at least 30% of the population needs to be identified to draw robust results from social network analysis (Silk et al., 2015). In Marley, this condition was easily satisfied (40–50%), but across the entire wood our sample was only just on this threshold (30%). If a greater proportion of individuals throughout Wytham had a known personality, we would have expected to detect significant positive assortment throughout the woodland. Our study therefore highlights the importance of knowing the spatial distribution of individuals sampled within a population, as unequal sampling can impact analyses.

The detection of positive assortment in the nearest-neighbour networks, but not those generated using the Thiessen polygon approach, is a notable finding with broader methodological and biological implications. This result may be due to the various assumptions involved in using Thiessen polygons to model animal territory boundaries, such as a spatially contiguous area and nonoverlapping territories (Appendix 3). Indeed, it is probable that territories do overlap, especially in areas of high settlement density. Thiessen polygon networks assume that competition between neighbours sharing a boundary determines territory formation (Schlicht et al., 2014) but in very clustered regions such as Marley, it is unlikely that birds only interact with their adjacent neighbours. In fact, great tits in this population are known to move beyond the territory boundaries predicted from Thiessen polygons (Cole, Morand-Ferron, Hinks, & Quinn, 2012). Indirect interactions through singing may also influence male territory choice, especially since birdsong has been shown to reveal individual personality (Garamszegi, Eens, & Török, 2008). Furthermore, birds living in relatively dense areas are also likely to hear the song of neighbours with whom they do not share a territory boundary and so may still be familiar with more distant neighbours (Briefer, Rybak, & Aubin, 2010). Notably, a recent study exploring song traits in wild territorial great tits also found that results varied depending on whether the analysis was conducted in relation to the spatial proximity of breeding individuals versus those sharing a territory boundary (Snijders, van der Eijk, van Rooij, de Goede, van Oers, & Naguib, 2015). They therefore concluded that neighbourhood structuring may be an important factor during the breeding season. In a similar way, the results of our analyses suggest that it is the personality of individuals in the local neighbourhood, rather than only adjacent territory holders, that contributes to the pattern of assortment in the nearest-neighbour networks.

Additional statistical analyses were conducted to understand what drives this pattern of spatial assortment. If nestboxes were consistently occupied by birds of certain personality types, this would suggest that where an individual chooses to breed was partly dependent on the surrounding ecological environment. However, there was no evidence that nestboxes were repeatable in terms of the personalities of the occupants. Neither was there evidence that bolder males chose to nest in areas with a higher or lower density of occupied territories, indicating that the observed assortment by personality was not clearly driven by differences in local population densities. Also relevant to this study, there is no evidence in this great tit population of assortative mating with respect to personality traits (Patrick et al., 2012).

Overall, our analyses suggest that social processes may be more important than environmental variation in determining social structure of a breeding great tit population with respect to personality. The finding that males, but not females, show significant assortment may reflect the role that personality plays in male territory choice and establishment, with shy males less likely to nest near bolder, more aggressive individuals. Female breeding location, however, may depend on other factors, such as mate choice, rather than the behavioural traits of surrounding females. Since only males show positive assortment by personality type in the winter foraging flocks (Aplin et al., 2013), this difference in assortment between the sexes may be a result of social ties formed during winter that have persisted into the breeding season. Recent findings reveal that social associations in winter foraging flocks can carry over into patterns of nestbox prospecting as the breeding season begins (Firth & Sheldon, 2015). In fact, stronger associations during the winter relate to closer nesting proximities in the subsequent breeding season, even more so than expected from winter spatial locations alone (Firth & Sheldon, 2016). Establishing a territory near a familiar individual may reduce aggressive encounters, leading to fewer costly interactions (Temeles, 1994). In addition, neighbour familiarity has been shown to have a positive influence on cooperative interactions and reproductive success in this great tit population (Grabowska-Zhang et al., 2012a, Grabowska-Zhang et al., 2012b). Understanding the relative importance of social versus spatial effects in structuring populations (Farine et al., 2015, Firth and Sheldon, 2016, Shizuka et al., 2014) and shaping population processes (Firth, Sheldon, & Farine, 2016), particularly with respect to the personality of individuals, is a key issue highlighted in this study which warrants further investigation.

The tendency for positive assortment by personality among males during the breeding season has implications for the way that selection is likely to act on behavioural phenotypes. The nature of interactions between territorial neighbours may differ considerably depending on their personalities. Shy great tits tend to respond less strongly to social confrontation than bold individuals, in terms of both territorial intrusion and approaches from the opposite sex (Amy et al., 2010, Carere et al., 2005, Snijders et al., 2015b). A potential consequence of this spatial assortment is that fights during the establishment and maintenance of territory boundaries may be less intense or less frequent between shyer individuals. Indeed, there is evidence of a positive correlation between exploration behaviour and singing intensity in captive great tits (Naguib, Kazek, Schaper, van Oers, & Visser, 2010), indicating that bold males invest more in territorial defence and attracting mates (Catchpole & Slater, 2008). Although bold territorial neighbours may suffer the costs of more intense interactions, they might also gain a shared benefit through more effective repulsion of common threats, such as conspecific intruders. However, bold individuals also tend to exhibit risk-prone behaviour, potentially increasing their exposure to predation (Bell et al., 2013, Cole and Quinn, 2014, Quinn et al., 2012).

Our findings also have wider relevance to previous research on this great tit population, which showed that bold males were more likely to engage in extrapair copulations (Patrick et al., 2012). It was suggested that this pattern may arise from breeding site choice if bolder males bred in more densely populated areas with higher encounter rates (Patrick et al., 2012). However, in our study we found no evidence for a correlation between personality and breeding density. Instead, the results reported here indicate that this observed association between personality and extrapair paternity may be driven largely by behavioural differences. In addition, given that bold great tits tend to be more promiscuous, and our finding that they are more likely to breed near other bold males, the incidence of extrapair paternity may be expected to be spatially clustered. This could have implications for male–male cooperation, as evolutionary modelling suggests that paternity uncertainty may incentivize males to form cooperative alliances and reduce their territorial aggression (Eliassen & Jørgensen, 2014).

The results of this study encourage further research into the role of animal personalities in structuring populations and the evolutionary consequences. Following from the evidence presented here that territorial males show positive assortment with respect to personality, an essential question to address is whether the fitness consequences of an individual's personality are not simply a function of its own phenotype but also dependent on the personalities of its neighbours. Thus, if the strength and direction of selection on behavioural phenotypes varies according to an individual's social environment, this may help explain the puzzle of the maintenance of personality variation within animal populations. Previously, evolutionary game theory has been used to account for this observation, with individuals gaining higher fitness payoffs if their strategy is rare (Dall et al., 2004). Although such negative frequency-dependent selection on personality is likely to play a part in maintaining this variation, the nonrandom distribution of behavioural phenotypes within social networks highlights the importance of also considering the role of social selection.

### Conclusions

Through examining the social structure of a wild bird population, we show that males are positively assorted by behavioural phenotype and are more likely to breed closer to other males of similar personality. This adds to previous studies of behavioural assortment in nonterritorial populations by revealing that individuals may also associate with those of like personality during the breeding season. This novel finding has implications for sexual and social selection. Studies of the relationship between social network structure and personality in breeding populations of other vertebrate taxa should be conducted to determine the generality of this finding.

## Conflict of interest

The authors declare no conflict of interest.

## Figures and Tables

**Figure A1:**
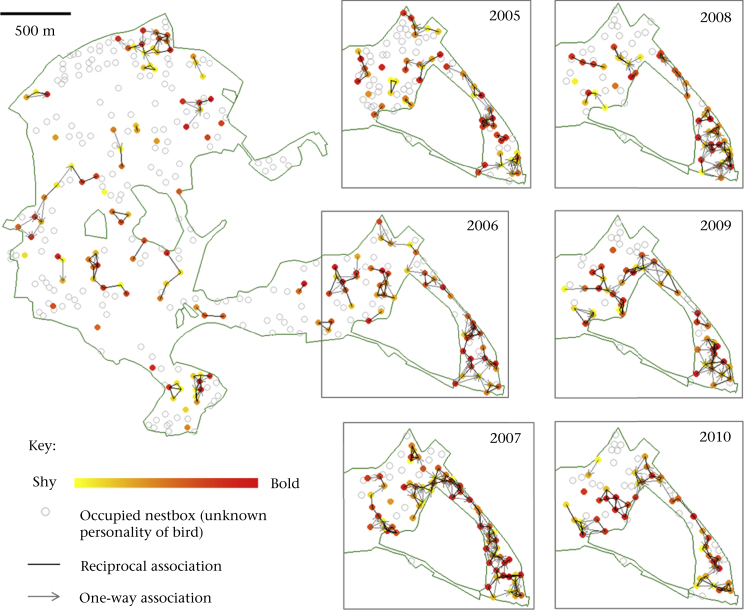
Female social networks (five nearest neighbours) in Wytham's great tit population during the breeding season. A representative network for Wytham is depicted (2006), along with the Marley networks for each year (2005–2010). Coloured nodes denote individuals with a personality score and open circles mark nestboxes occupied in that year but where the female's personality is unknown. Black lines denote a two-way association (both each other's nearest neighbours), whereas grey arrows depict the direction of one-way associations. For these directed networks, 70–80% of all links are reciprocal.

**Table A1:** Results of assortativity analyses on binary social networks within Marley using the nearest-neighbour (NN) method

Network type	Male					Female				
	No. of links	Mean assortment		Effect size	*P*	No. of links	Mean assortment		Effect size	*P*
		Observed	Permuted				Observed	Permuted		
3-NN	60.5	0.139	−0.032	0.171	**0.010**	93.2	0.004	−0.023	0.027	0.592
5-NN	102.7	0.086	−0.023	0.110	**0.040**	153.2	0.044	−0.022	0.065	0.128
7-NN	141.8	0.105	−0.026	0.131	**0.002**	212.0	0.049	−0.022	0.071	0.066

The observed and null assortment scores (averaged across all 6 years) are based on binary networks, with values in bold indicating significance at *α* = 0.05. The permuted assortment represents the mean of 1000 randomizations according to the null model.

**Table A2:** Results of assortativity analyses on weighted social networks within Marley using the nearest-neighbour (NN) method (duplicated associations removed)

Network type	Male					Female				
	No. of links	Mean assortment		Effect size	*P*	No. of links	Mean assortment		Effect size	*P*
		Observed	Permuted				Observed	Permuted		
3-NN	58.4	0.144	−0.032	0.175	**0.014**	90.6	0.001	−0.024	0.025	0.654
5-NN	98.7	0.090	−0.028	0.118	**0.032**	146.3	0.031	−0.022	0.053	0.230
7-NN	135.6	0.106	−0.027	0.134	**0.006**	200.6	0.034	−0.022	0.056	0.138

The observed and null assortment scores (averaged across all 6 years) are based on weighted networks, with values in bold indicating significance at *α* = 0.05. The permuted assortment represents the mean of 1000 randomizations according to the null model. Repeated links across years were deleted over 1000 randomizations and the median *P* value is reported (since this proved most robust to type I and II errors).

**Table A3:** Results of assortativity analyses on weighted social networks within Marley for individual years using the nearest-neighbour (NN) method

Network type	Year	Male					Female				
		No. of links	Assortment		Effect size	*P*	No. of links	Assortment		Effect size	*P*
			Observed	Permuted				Observed	Permuted		
3-NN	2005	49	0.254	−0.039	0.293	0.128	75	0.176	−0.019	0.194	0.230
	2006	78	0.027	−0.023	0.049	0.718	78	−0.144	−0.026	−0.117	0.452
	2007	89	0.126	−0.030	0.156	0.282	155	0.016	−0.022	0.038	0.702
	2008	59	−0.024	−0.031	0.007	0.938	78	−0.178	−0.028	−0.150	0.328
	2009	57	0.401	−0.040	0.442	**0.026**	90	−0.148	−0.032	−0.116	0.400
	2010	31	0.123	−0.034	0.157	0.466	83	0.302	−0.030	0.332	**0.032**
5-NN	2005	82	0.086	−0.027	0.117	0.396	115	0.204	−0.026	0.230	0.056
	2006	125	0.038	−0.035	0.073	0.492	139	−0.061	−0.020	−0.041	0.724
	2007	151	0.107	−0.026	0.132	0.238	251	0.025	−0.014	0.039	0.656
	2008	108	−0.054	−0.024	−0.031	0.854	133	−0.013	−0.022	0.009	0.878
	2009	96	0.291	−0.031	0.322	**0.024**	140	−0.130	−0.024	−0.106	0.352
	2010	54	0.126	−0.043	0.169	0.338	141	0.256	−0.023	0.279	**0.032**
7-NN	2005	120	0.072	−0.032	0.105	0.336	166	0.197	−0.022	0.219	0.052
	2006	170	0.093	−0.024	0.117	0.240	195	−0.011	−0.021	0.010	0.860
	2007	209	0.131	−0.024	0.154	0.098	344	0.024	−0.014	0.038	0.532
	2008	142	−0.021	−0.026	0.005	0.902	180	−0.055	−0.022	−0.033	0.786
	2009	137	0.208	−0.032	0.240	**0.036**	196	−0.054	−0.022	−0.032	0.820
	2010	72	0.165	−0.038	0.202	0.204	191	0.211	−0.024	0.235	**0.026**

The observed and null assortment scores are based on weighted networks, with values in bold indicating significance at *α* = 0.05. The permuted assortment represents the mean of 1000 randomizations according to the null model. In most cases the observed assortment score is greater than that generated from the null model (i.e. a positive effect size).

**Table A4:** Average distances between connected individuals in the nearest-neighbour (NN) networks for Marley

Network type	Year	Average distance (m) ± SE	
		Male	Female
3-NN	2005	50.9±3.1	51.6±2.4
	2006	61.3±2.6	62.9±3.1
	2007	53.4±2.1	53.1±1.6
	2008	55.0±3.1	51.4±2.9
	2009	60.4±3.9	64.2±3.1
	2010	60.1±4.3	54.4±2.8
5-NN	2005	62.0±3.4	61.0±2.9
	2006	73.4±2.6	77.4±3.0
	2007	65.2±2.3	63.6±1.7
	2008	67.6±2.7	63.0±2.7
	2009	75.8±4.0	76.3±3.0
	2010	73.7±4.8	68.4±2.9
7-NN	2005	74.5±4.2	71.7±3.1
	2006	83.9±2.8	88.3±2.9
	2007	75.0±2.3	73.1±1.8
	2008	75.8±2.9	72.2±2.8
	2009	88.6±3.9	88.4±3.2
	2010	82.0±4.5	80.1±3.1

**Table A5:** Results of assortativity analyses on weighted social networks within Wytham using the nearest-neighbour (NN) method

Network type	Male					Female				
	No. of links	Mean assortment		Effect size	*P*	No. of links	Mean assortment		Effect size	*P*
		Observed	Permuted				Observed	Permuted		
3-NN	137.2	0.041	−0.011	0.053	0.250	184.8	0.020	−0.011	0.031	0.424
5-NN	231.3	0.049	−0.011	0.060	0.082	311.3	0.022	−0.010	0.032	0.328
7-NN	316.5	0.065	−0.011	0.076	**0.018**	433.8	0.028	−0.010	0.038	0.142

The observed and null assortment scores (averaged across all 6 years) are based on weighted networks, with values in bold indicating significance at *α* = 0.05. The permuted assortment represents the mean of 1000 randomizations according to the null model.

**Figure A2:**
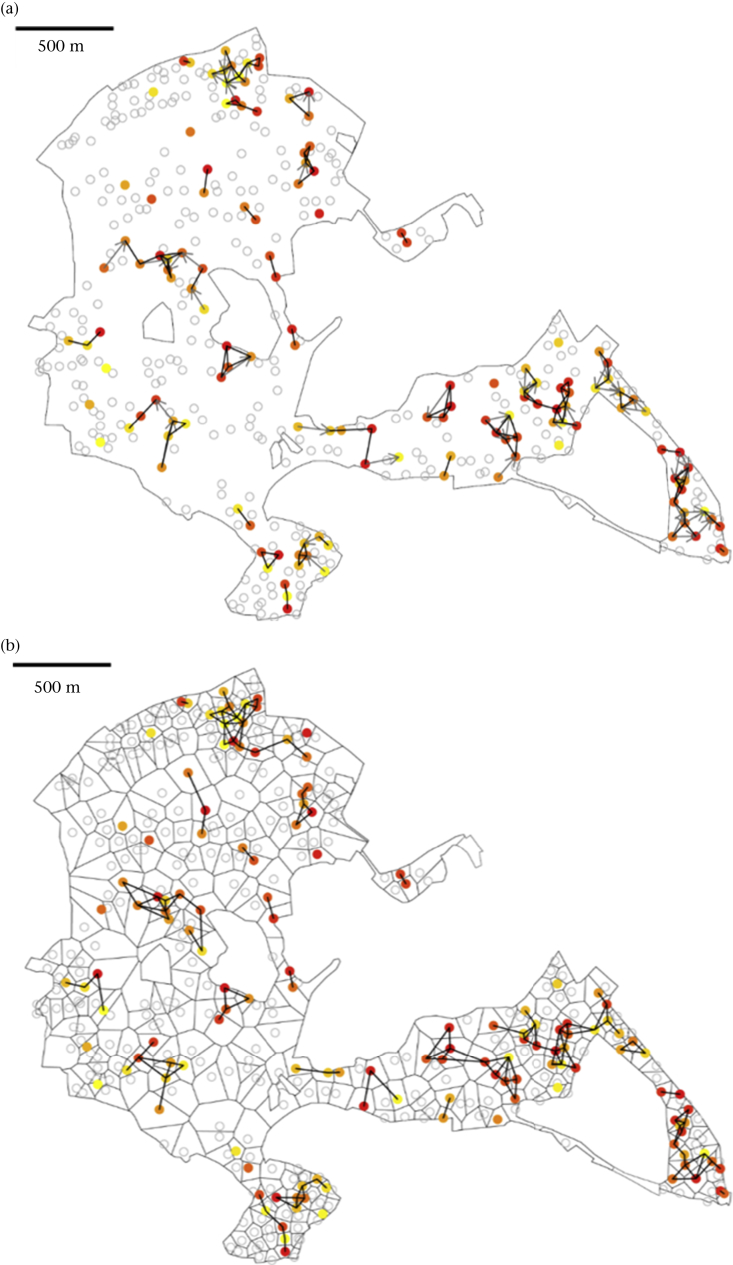
Comparison of male social networks in Wytham's great tit population generated using the (a) 5-nearest-neighbour method and (b) Thiessen polygon approach for a representative year (2006). Coloured nodes denote individuals with a personality score and open circles mark nestboxes occupied in that year but where the male's personality is unknown. Black lines in (a) denote a two-way association (both each other's nearest neighbours), whereas grey arrows depict the direction of one-way associations. The Thiessen polygons in (b) mark the predicted territory for each breeding pair, with black lines linking males with a shared boundary.

## Appendix 3 Comparison of nearest-neighbour and Thiessen polygon networks.

We note that in contrast to the nearest-neighbour networks, those generated using Thiessen polygons to estimate territory boundaries did not suggest any significant assortment by personality type. This is an interesting finding which may be due to a number of different factors. Unlike the directed networks generated by the nearest-neighbour approach (Appendix Fig. A2a), those constructed using the Thiessen polygon method are undirected since any individuals that share a territory boundary are connected in the network (Appendix Fig. A2b). There are also several assumptions associated with using Thiessen polygons to predict animal territories such as having a contiguous study area and discrete territories (Schlicht et al., 2014). However, it is likely that territories overlap, particularly in the Marley section of the woods which has a high settlement density. Since it is this region that drives the pattern of assortment (due to the higher proportion of individuals with a personality score), perhaps the Thiessen polygon method does not work well for areas that are highly clustered with occupants. Indeed, the predicted territories in Marley are very small (Appendix Fig. A2b) and so it is unlikely that the birds only interact with their immediate neighbours. An inherent assumption of this Thiessen polygon method is that competition with adjacent neighbours governs territory formation (Schlicht et al., 2014) but there are likely to be additional mechanisms involved that can transcend territory boundaries such as song production or individual movement. Also, in other areas of the wood with a low density of occupied nestboxes, this Thiessen polygon approach tends to overestimate territory size (Schlicht et al., 2014), leading to some unrealistically large territories. As a result, numerous associations in these networks seem rather improbable given the considerable distance between some nestboxes.
